# Calcined Clay as Supplementary Cementitious Material

**DOI:** 10.3390/ma13214734

**Published:** 2020-10-23

**Authors:** Roman Jaskulski, Daria Jóźwiak-Niedźwiedzka, Yaroslav Yakymechko

**Affiliations:** 1 Faculty of Civil Engineering, Mechanics and Petrochemistry, Warsaw University of Technology, Łukasiewicza 14, 09-400 Płock, Poland; Yaroslav.Yakymechko@pw.edu.pl; 2 Institute of Fundamental Technological Research, Polish Academy of Sciences, Pawińskiego 5b, 02-106 Warsaw, Poland; djozwiak@ippt.pan.pl

**Keywords:** calcined clay, binder, supplementary cementitious materials, cement-based materials

## Abstract

Calcined clays are the only potential materials available in large quantities to meet the requirements of eco-efficient cement-based materials by reducing the clinker content in blended cements or reducing the cement content in concrete. More than 200 recent research papers on the idea of replacing Portland cement with large amounts of calcined clay are presented and discussed in detail. First, the fundamental information about the properties and structure of clay minerals is described. Then, the process of activation and hydration of clays is discussed, including the methods of pozzolanic activity assessment. Additionally, various testing methods of clays from different worldwide deposits are presented. The application of calcined clay in cement and concrete technology is then introduced. A separate chapter is devoted to lime calcined clay cement. Then an influence of calcined clay on durability of concrete is summarized. Finally, conclusions are formulated.

## 1. Introduction

Sustainable development, understood as progress that meet the needs of the present without compromising the ability of future generations to meet their needs, is now the basic idea behind the current paradigm of technological progress. The greatest threat to the achievement of the objectives of sustainable development is the excessive emission of greenhouse gases, including, above all, carbon dioxide, which results in global warming with many serious negative consequences for future development and its prospects.

The building industry makes a significant contribution to increasing the carbon dioxide content in the atmosphere, with cement production accounting for 5% of global carbon dioxide emissions [1] being its largest source. This is due, on the one hand, to the high energy intensity of the Portland clinker production process. The production of one ton of clinker requires the supply of 3.1–3.8 GJ of heat, whereas in the older generation wet method kilns the demand may reach even 6 GJ/t [1]. On the other hand, during cement production, carbon dioxide is emitted from raw materials, mainly limestone. The amount of this emission is about 0.53 kg/kg of clinker [1].

The possibilities of reducing the energy intensity of cement production are limited, although it is already possible to reduce heat consumption to 2.9 GJ/t of clinker. The use of alternative fuels further reduces carbon dioxide emissions from fossil fuels. However, the use of limestone in clinker production cannot be eliminated or reduced. Therefore, solutions to further reduce CO_2_ emissions to the atmosphere by the cement and concrete industry are the production of blended cements that reduce the amount of clinker and the use of supplementary cementitious materials (SCM), mainly pozzolans, in the concrete technology. A pozzolan, according to ASTM C125, is “a siliceous and aluminous material which, in itself, possesses little or no cementitious value but which will, in finely divided form in the presence of moisture, react chemically with calcium hydroxide at ordinary temperature to form compounds possessing cementitious properties” [2].

The most commonly used SCM so far is fly ash from coal power plants. However, this additive, which is used in the production of both cement and concrete, is slowly losing its primary importance due to the progressive decommissioning of coal and lignite power plants. As a result, the supply of fly ash will be significantly reduced in the near future. Another cement additive used to reduce clinker content is a granulated blast furnace slag. However, it has no potential to replace fly ash, not least because its global supply is far below potential demand. Additionally, it is only available in countries where the steel industry exists. However, even where it is available, its share in blended cements production is not large. In India, for example, Portland slag cement accounts for only about 8% of total cement production, while ordinary Portland cement accounts for 24% of production, and Portland pozzolana cement accounts for 65%, where the main addition is fly ash [3,4].

The decreasing supply of fly ash from the power industry is encouraging the search for new sources of pozzolan additives for cement and concrete production. One of the directions of this search is ashes obtained from biomass combustion, [5]. Another is the use of natural and artificial pozzolana. The former include, among others, volcanic tuffs already known in ancient times or zeolites. The best known representative of the second group is the metakaolin formed by the calcination of kaolinite from clays with its significant content. 

Metakaolin was shown to be the best clay raw material for SCMs production, but in its pure form it is only found in a limited number of deposits, so its availability is not sufficient to meet the needs of the building materials industry. It is also in the focus of interest of other industries [6,7]. For this reason, there has been interest in the possibility of producing SCMs from other locally available natural clays containing, in addition to kaolinite, other minerals which have the potential to develop pozzolanic activity upon appropriate activation. Research conducted in this direction led to the separation of a new group of pozzolanic materials—calcined clay.

Clay is a widely spread material in the world, cheap and easily accessible [8]. At the same time, it is a material with a great diversity in terms of mineralogical composition, hence numerous literature items devoted to the analysis of the possibility of using clays from specific deposits for the production of SCM in the calcination process [9,10,11].

In general, the literature on the activation of clay minerals and their use in civil engineering is very rich and covers a wide range of issues. It would be very difficult to discuss this vast area of research in one review article, so certain assumptions on the scope of this review were made First of all, it focuses on calcined clays used as SCM in binders, where cement is the dominant component, and in the cement-based concrete. Outside the scope of interest remain cement-free binders based on calcined clays, which are the basis of geopolymers, as well as other alkaline-activated binders (including clay minerals). Issues that do not fall within the assumed topics of the article are mentioned where it was indicated for various reasons.

The paper is divided into eight thematic sections. The first section describes a fundamental knowledge about clay minerals and publications on clays from various deposits in the world. In the next section the topic of clay minerals activation with emphasis on calcination process and research of hydration mechanisms and pozzolanic activity with factors influencing them are presented. A separate section is devoted to the role of calcined clays in concrete technology (as SCM) and in blended cements production. Due to the wealth of literature devoted to lime calcined clay cement (LC^3^), a separate section is also devoted to it. The next chapter discusses the results of research on the impact of calcined clays on concrete durability. Numerous publications on calcined clay, which cannot be clearly classified as one of the above thematic areas, have been placed in a separate section devoted to suitable aspects of the application of these materials. The summary presents the conclusion of the literature review

## 2. Characteristic of Clay Minerals

**Figure 1:**
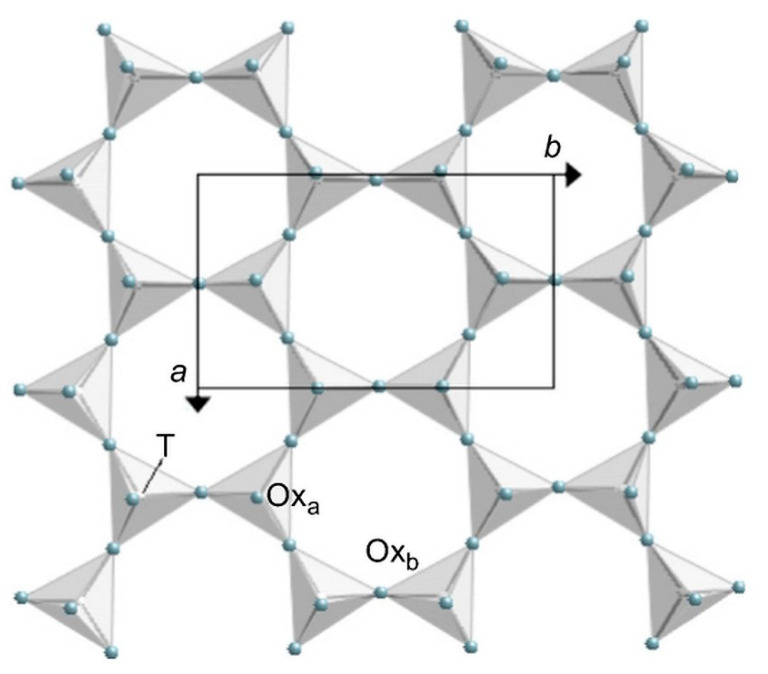
The tetrahedral sheet. T, tetrahedral cations; O*xa*, apical oxygen atoms; O*xb*, basal oxygen atoms. *a* and *b* refer to unit cell parameters. Reprinted from Brigatti, M.F.; Galán, E.; Theng, B.K.G. Structure and Mineralogy of Clay Minerals. In *Handbook of Clay Science*; Bergaya, F., Lagaly, G., Eds.; Elsevier Ltd: Amsterdam, The Netherlands, 2013; pp. 21–81. License number 4912071405833.

**Figure 2:**
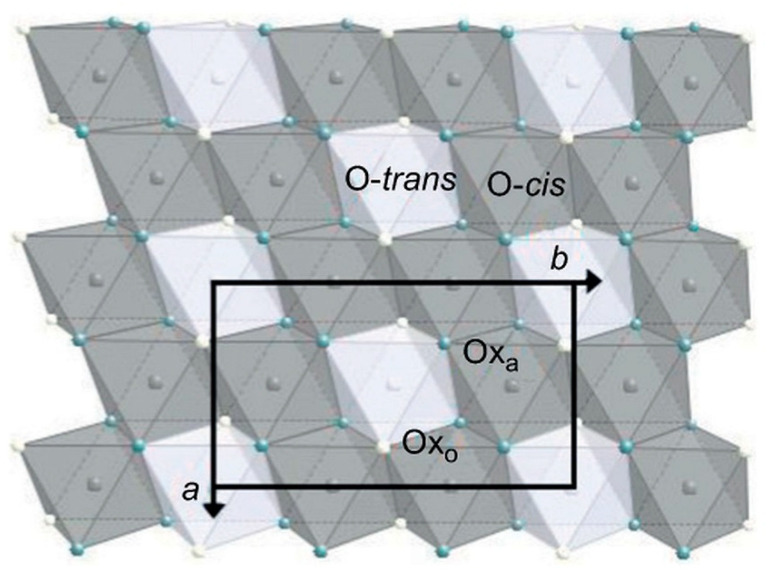
The octahedral sheet. O-*trans*, *trans*-oriented octahedra; O-*cis*, *cis*-oriented octahedra; O_xa_, apical oxygen atoms; O_xo_, OH, F, Cl octahedral anion. *a* and *b* refer to unit cell parameters. Reprinted from Brigatti, M.F.; Galán, E.; Theng, B.K.G. Structure and Mineralogy of Clay Minerals. In *Handbook of Clay Science*; Bergaya, F., Lagaly, G., Eds.; Elsevier Ltd.: Amsterdam, The Netherlands, 2013; pp. 21–81. License number 4912071405833.

Clay minerals, that are the subject of this review, belong to two main groups, which are often referred to in the literature as 1:1 and 2:1 minerals. These two terms result from their structure, which consists of repetitive tetrahedral (T) and octahedral (O) layers [12]. The T layer consists of corner-contacting tetrahedral Si^4+^, Al^3+^, and Fe^3+^ cations, while the O layer consists of edge-contacting octahedral Al^3+^, Fe^3+^, Mg^2+^, and Fe^2+^ cations, which are arranged alternately in *cis* and *trans* configurations (see Figure 1 and Figure 2). 

At the contact of T and O layers there is a layer of oxygen atoms (so-called apical oxygen). Additionally, the O layer contains oxygen atoms (non-apical oxygen) forming OH^−^ groups and other anions (F^−^, Cl^−^), which are located in the corners of octahedrons, but are not shared with the T layer [12]. 

**Figure 3:**
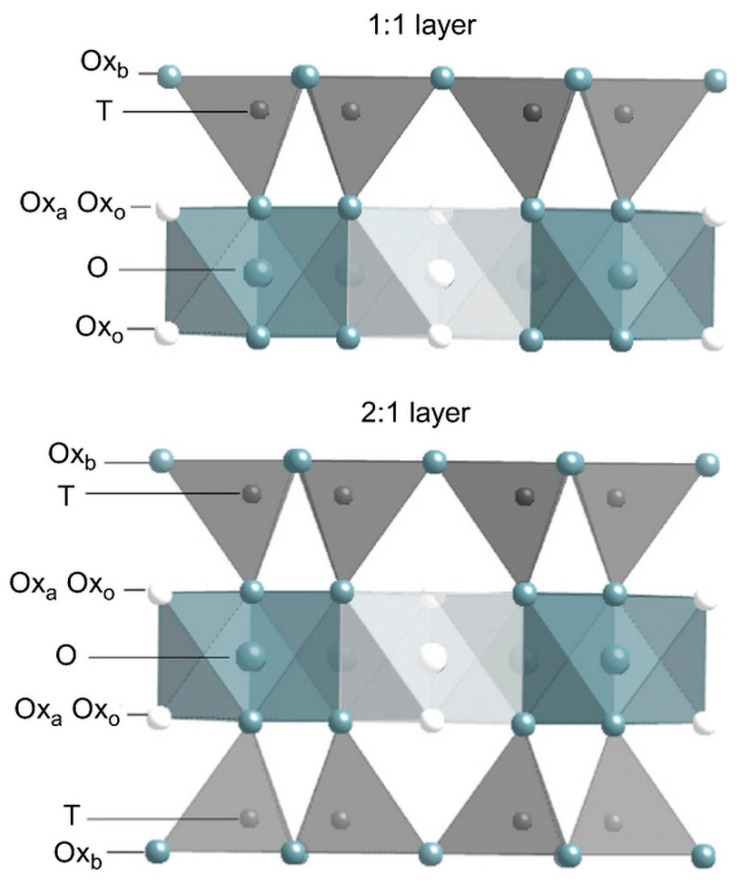
Models of 1:1 and in 2:1 layer structures. O_xb_, basal oxygen atoms; T, tetrahedral cations; O, octahedral cations; O_xa_, apical oxygen atoms; O_xo_, octahedral anions (OH, F, Cl). Reprinted from Brigatti, M.F.; Galán, E.; Theng, B.K.G. Structure and Mineralogy of Clay Minerals. In *Handbook of Clay Science*; Bergaya, F., Lagaly, G., Eds.; Elsevier Ltd.: Asmetdam, The Netherlands, 2013; pp. 21–81. License number 4912071405833.

The division of minerals into 1:1 (TO) or 2:1 (TOT) groups results from the repetitive arrangement of layers of the one or the other type (see Figure 3). This division is far from covering the rich diversity of clay minerals, at least because 2:1 minerals may additionally contain anhydrous interlayer cations, hydrated interlayer cations or octahedral interlayer sheet. In addition, clays also contain complex minerals in which TO and TOT layers co-exist and can be distributed regularly or randomly. 

Minerals 1:1 include two groups: the kaolin group and the serpentine group. The first group includes: kaolinite, dickite, nicrite, halloysite, hisingerite. To the second group belong, among others, lizardite, antigorite, chrysotile, caryopilite, pyrosmalite, polygonal serpentines and polyhedral serpentines. 

Minerals 2:1 form a large group of which the following should be mentioned: pyrophyllite and talc both of ideally layered structure; mica, more than 200 variants [13], of which muscovite and illites will be discussed further on; and smectites, of which montmorillonite is discussed further on. The 2:1 structure also has vermiculite, chrorites and, finally, minerals with mixed layer structure, such as illite-smectite [14,15], chlorite-smectite [16] or illitic-chlorite. The calcination of the former is devoted, among others, in the publication of Garg and Skibsted [15].

A more detailed discussion of the properties and structure of clay minerals is beyond the scope of this review. In the following section, the minerals which are part of calcined clays and which determine their properties are discussed in more detail. The discussion is focused on their properties, which are important in the activation process and determine their properties as pozzolan materials. The article describes also factors influencing the optimization of activation process. 

The most important clay minerals, which are subject to temperature activation and, therefore, are discussed in more detail in this paper, are kaolinite and montmorillonite, which also occurs in the form of variants called Ca-montmorillonite and Na-montmorillonite [17,18,19]. Additionally, illite, being a poorly crystallised mica, and muscovite belonging to the same family, can be subjected to the calcination process, although both show low pozzolan activity even after heat treatment (illite, however, slightly higher than muscovite). Nevertheless, both are also included in this review of clay minerals. Relatively little attention is paid to sepiolite, halloysite and mixed-layer minerals in the research on pozzolan activity, therefore, due to a small number of publications, these minerals are discussed more briefly in this paper.

Kaolinite and the product of its calcination, i.e., metakaolinite, are the most thoroughly tested of the clay minerals that are part of the clays subjected to calcination in order to use them as SCMs due to their pozzolanic activity. Part of this literature can be found in [20,21]. Other minerals are much less popular so far, so Garg and Skibsted’s publications on montmorillonite are worth mentioning here [22,23]. Garg and Skibsted [22] presented the result of investigation on the activity of calcined pure montmorillonite due to the changes in the structure under the heating. The authors traced the changes in the structure of montmorillonite heated up to 1100 °C using the NMR technique. The results indicate that montmorillonite exhibits pozzolanic activity both in the raw state as well as heated up to any temperature value it was subjected to in the mentioned studies, although above 800 °C a clear decrease in this activity is visible. 

It is not only montmorillonite that exhibits pozzolanic activity in its raw state. Among such minerals we can also include illite, kaolinite and muscovite [24,25,26]. However, it is montmorillonite that is most active in the raw state. The least active of them is muscovite due to a small proportion of amorphous phase, which is additionally accompanied by very high water content [27]. Nevertheless, it is commonly accepted that the most active mineral of the pozzolanic is metakaolinite. The study of He et al. [17] revealed that Ca-montmorillonite may show even higher pozzolanic activity after calcination. It is associated with a high content of amorphous silica. Additionally, in [28] two types of bentonite (containing mainly montmorillonite) were tested and obtained better results than most of the five kaolinites, although it should be added that such results were obtained only in lime consumption and electric conductivity tests.

To the group of kaolinites, i.e., minerals 1:1 belongs halloysite—a lesser known close “cousin” of kaolinite. Both these minerals have identical chemical structure with the only one difference that halloysite may contain two additional water molecules in the interlayer space. Halloysite is, therefore, sometimes referred to as hydrated kaolinite. This small difference has significant consequences for the structure of this mineral, which forms different forms such as tubes, spheres and laths [12,29]. Water in the interlayer space can be easily removed by thermal treatment and such dehydrated halloysite is sometimes referred to as metahalloysite. The results of a study on the pozzolanic activity of clays rich in halloysite are presented in papers of Tironi et al. [30,31]. Obtained results confirmed the high pozzolanic activity of calcined clays containing halloysite, confirming their usefulness as a raw material for SCM production. 

Muscovite is also included among the clay minerals that show pozzolanic activity after appropriate heat treatment, although in this case the results are ambiguous. Ambroise [24], by examining the possibility of activation at 750 °C of, among others, muscovite and phlogopite, obtained results indicating low effectiveness of this form of treatment. Samples of calcined muscovite blended with calcium hydroxide after seven days of curing in the moulds disintegrated after placing them in water, where they were to undergo further curing. On the other hand, [8] showed that muscovite increases the hydration heat of the cement and its effect varies depending on the temperature of thermal treatment (the raw material was tested, as well as heated at various temperatures from 500–950 °C). Additionally, in [27] the calcined muscovite pozzolanic activity at 800 °C was shown, although it was relatively low compared to calcined illite and kaolinite. 

Another clay mineral which has been studied due to its potentially pozzolanic properties is sepiolite [17,32]. It is a 2:1 group mineral with an unusual structure due to discontinuity of TOT layers [12]. Studies have shown that without heat treatment sepiolite is not a material that is active as a pozzolan. Its additional disadvantage, from a technological point of view, is high water demand. The use of sepiolite in raw form as a partial substitute for cement led to a significant decrease in the strength of the mortar used to assess the hydraulic properties of potentially pozzolanic materials. On the other hand, calcination carried out at the temperature of 830 °C allows to obtain a material with distinct, although relatively low pozzolanic activity and significantly reduced water demand [32]. The low pozzolanic activity of sepiolite may result from low Al content (about 1%), which, together with Si, is responsible for pozzolanic activity of clay minerals. 

Illit is one of the minerals which show practically no pozzolanic properties in their raw state. Heat treatment carried out at high temperatures (930–950 °C) both pure mineral with small admixtures [33] as well as clays with a dominant share of illite [34] leads to a material with moderate pozzolanic activity. The dehydroxylation process itself, occurring at a lower temperature, does not lead to activation of the illite and formation of a material with a positive value of hydraulic index whereas, at the temperature that causes the destruction of its structure, a partial recrystallization of the resulting amorphous phase takes place at the same time, which reduces the potential pozzolanic activity of this mineral.

Mixed-layer minerals, which are characterised by the presence of different types of layers, are very common in clays. According to Weaver [35], it is estimated that out of 6000 tested samples of clay minerals from all over the USA as much as 70% of them contain different varieties of mixed-layer minerals. Such widespread minerals could not be omitted in studies on pozzolanic activity of clays, although the number of publications on this topic is small. He et al. [14], investigated the pozzolanic activity of synthetic clay consisting of layers of mica and smectite, used as a catalyst [36]. The share of illite (i.e., mica) in relation to montmorillonite (smectite) according to various studies of this material is in the range from 3:1 to 2:1. His studies showed that this mineral, after calcination at 960 °C, showed good pozzolanic properties, achieving in the test mortar strength of 113% in relation to mortar consisting only of OPC. 

The study of natural mixed-layer mineral of similar composition (illite/smectite in the ratio 70/30) was conducted by Garg and Skibsted [15]. In this case, the optimal calcination temperature was 900 °C and its exceeding led to crystallization of a part of amorphous phase and decrease of pozzolanic activity. Apart from a slightly different mutual ratio of mica and smectite, the mineral studied by Garg and Skibsted did not contain NH^4+^ ions, which may partially explain the discrepancy in the value of the optimal calcination temperature in relation to the findings of He et al.

Lemma et al. [16] study of mixed-layer clay consisting mainly of illite (45%) and chlorite (14%) containing also non-clay minerals, such as quartz (30%) and feldspar (10%), revealed high optimal calcination temperature of the material. The highest value of strength activity index (SAI) of this clay was observed after calcination at 1100 °C. More detailed analysis of the obtained results indicated that an increase in the pozzolanic activity at this temperature, in relation to that observed at 900 °C after total dehydroxylation of the illite, was caused by the transition of feldspar from a crystalline to an amorphous form. Although this mineral is not typical for clay rocks, its occurrence in the composition of clay intended for calcination may be a premise for carrying out this process at a higher temperature.

## 3. Activation and Hydration of Clays 

### 3.1. Activation Process and Its Conditionalities

Raw clays usually have a moderate or low pozzolanic activity. To increase it, they need to be activated. As a result of activation, clay minerals undergo processes of dehydroxylation and amorphisation and the accompanying change in coordination of Al ions. Those processes lead to, among other things, greater solubility of Al and Si ions and their greater reactivity, which is a basic condition for the demonstration of pozzolanic activity by clay minerals [37]. The activation process can be carried out mechanically (by grinding) [38,39,40] or thermally by heating to a temperature high enough to destroy the structure of the clay minerals, but low enough to avoid recrystallization and the formation of chemically inert phases. Some publications indicate that kaolinitic clays should be ground before they undergo calcination [41,42], i.e., their activation should have both mechanical and thermal component. Processes occurring during thermal or mechanical activation can also be induced chemically to some extent [37,43]. Sanchez et al. [44] studied the effects of thermal activation of kaolin supported by an activator in the form of 1% ZnO admixture. The efficacy of the activation process of acid treatment of clay before its calcination was also studied [45]. Due to the subject matter of this article, only thermal activation, i.e., calcination, will be described in more detail.

The effectiveness of the thermal activation process, and consequently pozzolanic activity of the obtained material, depends on many factors. These include: calcination temperature, particle size and shape, time and others [24]. The most attention is paid to the analysis of the temperature influence. According to Fernández et al. [18], the exposure of clay to too low or too high a temperature can significantly affect the activation process. In the former case, due to incomplete dehydroxylation, in the latter case, due to the melting of minerals and their subsequent recrystallization leading to the formation of phases which do not react with the cement hydration products and do not exhibit any pozzolanic activity. 

The process of dehydroxylation, i.e., tearing off OH^−^ groups [17], leads to serious damage in the crystal structure of clay minerals. As a result, among other things, it leads to increased exposure of Al ions on the surface of the mineral grains and increased solubility [18]. This effect is more pronounced in the case of kaolinite than in the case of group 2:1 minerals, which is reflected in the differentiation of pozzolanic activity of clay minerals. The structure of the minerals also affects the dehydroxylation temperature, which occurs in a wide range from 350 °C to 900 °C, and for its full effect in most cases the mineral must be heated to temperatures between 600 and 800 °C [46,47]. At temperatures below 600 °C, this reaction takes place only with kaolinite [48,49]. Montmorillonites undergo this process at temperatures between 550–850 °C. Illite is dehydroxylated in the temperature range 600–900 °C [18]. Neißer-Deiters et al. [8] have studied the influence of temperature on the properties of calcined mica consisting of muscovite with a small admixture of phengite. The investigated material was subjected to calcination at different temperatures ranging from 500 °C to 950 °C. As the raw muscovite already exhibits pozzolanic properties, the aim of the study was to determine whether and how the calcination influences its pozzolanic activity and water demand. The results indicate that the calcination process, regardless of the temperature, leads to slight changes in the above-mentioned parameters.

Another variable of importance in the thermal activation process is time. Its impact has been studied by Chakchouk et al. [50]. They determined the optimal parameters of mortar with the use of calcined clay. For this purpose a model with three variables was developed. In addition to the calcination time, the temperature of the calcination and the exchange rate of cement to calcined clay in the subsequently tested blended binder were also taken into account. In 23 individual experiments, the time varied from 1.32 h to 4.68 h. In most cases, it was 3 h. They showed, that a longer calcination time was beneficial in increasing the strength of the tested mortar if the temperature was lower than 700 °C, and above this temperature the effect was the opposite. 

The duration of the thermal activation process is usually counted in hours or minutes when using fluidized bed reactors [51]. This type of calcination is referred to as soak-calcination. This process is most commonly used and most of the publications are devoted to it, however, in these publications the time is sometimes given for the whole process, sometimes it is only the time of keeping the material at the highest temperature, and sometimes it is not specified at all. This makes it difficult an attempt to summarise and make clear recommendations. Especially since this time also depends on the degree of material fragmentation, its pre-treatment (e.g., drying to constant mass) or the type of device in which the activation is carried out (rotary kiln or fluidized bed reactor). 

However, regardless of whether the process of soak-calcination takes several minutes or several hours, the activation process known as flash-calcination, which lasts from fractions of a second to several seconds, can be clearly distinguished from it [51,52,53,54,55]. The shortening of the process results from a very large fineness of the processed material and from the high temperature in the calciner reaching 1100 °C. The combination of high pulverization and high temperature causes the clay subjected to calcination to be heated very quickly in its entire mass. The rise of temperature reaches 0.5–1.5 × 10^4^ °C/s [52,54]. Such a rapid temperature increasing leads to a material with slightly different characteristics from clay obtained from the soak-calcination process. In such clays in particular, the dehydroxylation process is sometimes not complete. Moreover, due to the rapid process, the obtained material has a lower density and higher porosity. This usually also translates into increased reactivity [51,53,54]. Most of the research on flash-calcination concerns clays with high kaolinite content, therefore, the work of Rasmussen et al. [53] is worth mentioning. They carried out a study using clays with a composition dominated by minerals from the 2:1 group. They concluded that, also in the case of this type of clays, flash-calcination can lead to the production of clays with a high-level of pozzolanic activity, and the advantage as compared to soak-calcination procedure in this case lies in the lower risk of melting and recrystallization of minerals, such as feldspar or spinel [53].

It is not only time, temperature or particle size that determine the success of the calcination process. Bich et al. [56] studied the conditions of the dehydroxylation process depending on the degree of ordering of the mineral structure. They used three clays with high kaolinite content and different degree of order of this mineral defined by values of P_0_ coefficient (it is defined further in the article). They showed, that in case of kaolinite with disordered structure (P_0_ = 0.68), that it was enough to heat the material at 650 °C for 45 min for full dehydroxylation whereas, in the case of kaolinite with well-ordered structure (P_0_ = 1.4), such time allowed dehydroxylation of 71–95% of kaolinite. The relationship between kaolinite dehydroxylation degree and lime consumption from CaO saturated solution after 28 days was also shown. Although the level of linear regression adjustment (R^2^ = 0.73) indicates that the kaolinite dehydroxylation level is an important but not the only factor shaping the calcined clay pozzolan activity even if it contains more than 75% of kaolinite.

The calcination process, due to its complexity resulting from a number of parameters regulating it, as well as from various possible variants of its execution, has also gained mathematical models describing it. Teklay et al. [54,57] developed two mathematical models of the calcination process. The first one [57], with reference to the material itself, models the calcined mineral in the form of spherical particles with specific physical parameters, which are affected by temperature, as a result of which some changes in its properties occur. This model is independent of the form of the process (flash- or soak-calcination). The other model [54] is an attempt to mathematically describe the flash-calcination process itself, allowing to determine its key parameters in relation to specific input data referring to the material subjected to heat treatment.

Among publications devoted to the activation of clays and clay minerals, there are also those concerning the conduct of this process with the use of additional substrates aimed at changing certain characteristics of the finished material. The primary purpose of such additives or admixtures is to increase pozzolanic activity [58]. As an example of research on the influence of admixtures on calcined clay, Taylor-Lange et al. [59,60] can be mentioned. The admixture in this case was zinc oxide, which was added to clays containing kaolinite, montmorillonite and illite both before and after calcination. The results were promising only in case of the clays containing kaolinite. The presence of calcined clay significantly reduced the delaying effect of zinc oxide on the cement hydration process. 

The increase in kaolinite pozzolanic activity was also obtained by Ghorbel and Samet [61], who added iron nitrate solution to clay rich in this mineral. As a result of calcination of such additionally enriched clay, hematite and goethite were formed. The authors came to the conclusion that the presence of the former increases the kaolinite pozzolanic activity as long as it does not exceed 2.7%. The presence of iron ions in clay subjected to calcination also has its less favourable effects. They are responsible for its reddish hue, which is visible in cements blended with use of such clay. The reddish shades of cement are sometimes improperly interpreted as a sign of poor quality of the material, hence the idea to study the calcination process in a reducing atmosphere [62]. In this process, ground petroleum coke was also added to the raw ground clay. As a result of such calcination, a product was obtained whose red colouring was much less intense. The durability of the obtained effect remains an issue to be investigated.

### 3.2. Activation of Various Clays and Clay Minerals 

It is assumed that the largest pozzolanic activity characterizes kaolinite, which also has the lowest activation temperature range. According to He et al. [17], calcination of this mineral can be successfully carried out even at 450 °C, although full completion of the dehydroxylation process requires a temperature of about 650 °C [63]. The high pozzolanic activity of calcined kaolinite results from the high content of hydroxyl groups and their location, which favours the exposure of Al groups on the surface of material grains after the dehydroxylation process [18]. Illite and montmorillonite also lose hydroxyl groups under the influence of temperature, but Al atoms remain in the structure of these minerals in places more difficult to access for cement hydration products, which may react with them [18]. As a result, their pozzolanic activity is lower than that of calcined kaolinite [24].

Metakaolinite, which is a product of kaolinite calcination, is a transition phase during the thermal transformation of kaolinite into mullite, which is an inactive pozzolanic mineral. Sperinck et al. [63] presents the process of kaolinite calcination modelled using the molecular dynamics (MD) simulations. The simulations were based on “heating” the simulated structure of the mineral to 1000 K, removing 10% of the initial number of hydroxyl groups and then quickly “cooling” it to 300 K in order to analyse the obtained results. The next steps of the simulation were carried out until all OH^−^ groups were removed. As a result of the simulation, a disordered mineral structure was obtained, in which the silicon layers showed little disorder, while the aluminium layers were significantly disordered and about 20% of the Al ions gained 5-fold coordination. It is less stable than the original six-fold coordination and four-fold coordination, which was adopted by most Al ions after calcination. This results in greater solubility and, as a consequence, also pozzolanic activity. Interestingly, the Al ions only moved within their own layer, as they were held back from further migration by a less affected silicon layer. 

Minerals of the 2:1 group are characterised by a fairly wide range of temperatures at which dehydroxylation occurs. As far as illite is concerned, the temperature required for this process is 650 °C, but further heating is possible (up to 930 °C), which leads to an increase in its pozzolanic activity, probably due to the progressive amorphisation of the structure. According to He et al. [17], Ca-montmorillonite and Na-montmorillonite undergo total dehydroxylation at temperatures of 730 °C and 740 °C, respectively. In the case of both these minerals, the pozzolanic activity increases also after heating at higher temperature, but not exceeding 930 °C. Differences in decarboxylation temperature between individual minerals of group 2:1, and even for the same mineral, were explained by Drits et al. in [64]. They indicated the role of *cis*-vacant (cv) and *trans*-vacant (tv) modified layers in determining this temperature. Both these variants of layers differ in the distance between the nearest hydroxyl groups. In tv 2:1 layers the bond length is 2.45 Å ± 0.05 Å, whereas in cv 2:1 layers it is considerably longer and is 2.85–2.88 Å. Hydrogen, which in the process of dehydroxylation passes from one group OH^−^ to another creating a water molecule, needs a little more energy in the latter case. This explains well the difference in temperature required for full hydroxylation between the 2:1 group minerals.

Studies on the process of montmorillonite calcination, using the NMR technique, were conducted by Garg and Skibsted [23]. They showed that up to the temperature of 200 °C the dehydration of the mineral takes place, then up to the temperature of 500 °C no significant changes occur and then between 500 °C and 600 °C the dehydroxylation process begins. This process lasts until the material reaches a temperature of 900 °C and is connected with the progressive amorphisation of the mineral structure. Starting from the temperature of 1000 °C, another process of changing the structure of the heated material begins, resulting in its recrystallization as a result of the final destruction of the original layered mineral structure. In addition to NMR studies, Gard and Skibsted [23] also studied the mineral’s pozzolanic activity by preparing mortars containing Portland cement and calcined clay at a ratio of 70:30 and analysing the products of hydration at specific intervals up to 1 year. The results indicated that 800 °C was the upper limit of the optimal calcination temperature range. The lower limit of this range was 750 °C. Interestingly, montmorillonite heated to a temperature above 850 °C showed significantly lower pozzolanic activity than in the raw state. The presented results are consistent with the results of Al and Si solubility tests carried out by the same authors [23]. Samples of calcined clay at two temperatures: 800 °C and 900 °C were subjected to 24-h treatment with 0.1 M NaOH. The solubility of Si and Al, which is a good indicator of pozzolanic activity, was about 4 times lower for clay activated at the higher of the given temperatures.

The less frequently occurring clay minerals, and thus slightly less popular among researchers, include pyrophylite, whose dehydroxylation processes and effects of thermal treatment are presented in papers [65,66]. Additionally, halloysite is not the subject of numerous publications [30,31], even though it is a clay mineral showing high pozzolanic activity after calcination. Moreover, it is interesting due to its differentiated structure. Its structure can form tubes or spheres, and these differences manifest themselves to a large extent in the pozzolanic activity of the calcined mineral. Halloysite in the spheroidal form is mainly active in the initial period due to more easily available reactive alumina. In the tubular form alumina cations are not so easy available, therefore, this form exhibits delayed activity [30].

Among the publications devoted to the study of clay activation, paper [67] should be mentioned, which covers the subject of activation of pure clays, among others kaolinite, illite, etc. Activation of clays containing smectites (bentonites) and kaolinite was studied by the authors of papers [68,69,70]. The analysis of activation process of clays containing illite and smectites (montmorillonite) in comparison to those containing kaolinite is included in [71]. Heat treatment of clays containing about 40% kaolinite as well as about 40% illite and montmorillonite is described in [49].

### 3.3. Tests of Eligibility of Clays for Activation Procedure

Since deposits of pure clays containing only one type of clay mineral are not rich enough to meet the growing demand of the construction industry for calcined clays as supplementary cementitious materials, it is important to assess the clays from existing deposits in terms of the possibility of using their resources as raw materials for the production of SCMs. The pozzolanic activity of clay is not a simple sum of the activity of its individual constituent minerals. The co-occurrence of some of them may give a synergy effect, but in other configurations the potential of any of the components may not be fully exploited. Hence the need to develop a methodology for the assessment of specific deposits arises in terms of their suitability for activation.

Diaz et al. [72] presented an idea for a method of initial assessment of clay based on its chemical composition. This method is primarily intended for the assessment of clays containing minerals from the kaolinite group, but its application is also possible for clays containing 2:1 minerals. In order to assess the suitability of clays from a particular deposit using this method, first of all their chemical composition should be determined, with the Al_2_O_3_ content, which should be more than 18%, then the ratio of Al_2_O_3_ to SiO_2_ content, which should be higher than 0.3 and, finally, the loss on ignition, which should not be less than 7%. Due to the deleterious effects of calcite and pyrite, the authors have added two additional conditions: CaO < 3% and SO_3_ < 3%. The authors are of the opinion that first of all, the exploitation should be carried out with the use of aluminium deposits containing over 40% of kaolinite. However, in order to take into account the pozzolanic activity of 2:1 group clays, they proposed a parameter defined as kaolinite equivalent calculated according to Equation (1):

%KEQ = {[m(350 °C) − m(850 °C)]/[m(200 °C) × 0.1396]} × 100,(1)

in which *m*(*x*) is the mass of the mineral after heat treatment at a given temperature *x*. 

A promising tool for a preliminary assessment of potential clay minerals’ pozzolanic activity may be cation exchange capacity, but not yet at this stage, as the author himself admits in his paper [73]. The development of an effective and simple methodology for the assessment of clay deposits for the cost-effectiveness of their exploitation as raw materials for SCMs production still remains an issue to be resolved.

### 3.4. Methods of Assessment of Pozzolanic Activity

The pozzolan reaction occurs between Al_2_O_3_-2SiO_2_ and Ca(OH)_2_, which in the case of concrete comes from the hydration of cement. The presence of water is also necessary for this reaction, as the reaction products are hydrates. As a result of the pozzolan reaction, CSH gel (CaO·SiO_2_·H_2_O) and hydrated calcium aluminates (e.g., C_4_AH_13_, C_2_AH_8_, C_3_AH_6_) are produced, as well as hydrated calcium aluminosilicates of the hydrogehlenite type C_2_ASH and hydrogarnets (C_3_AS_3_-C_3_AH_6_). Carboaluminates (e.g., calcium hemicarboaluminate hydroxide C_4_ACH_11_ which more detailed formula is C_3_A·0.5CaCO_3_·0.5Ca(OH)_2·_10.5H_2_O) may also be formed in the presence of carbon dioxide or limestone filler [47,74,75,76,77]. 

There are many different methods of determining pozzolanic activity. They are described among others in [28,58,78,79]. According to [78] they can be divided into direct and indirect ones. Direct methods include methods based on the analysis of Ca(OH)_2_ content and products of its reaction and changes in the content of these compounds over time. These are methods using XRD, TGA and DTA techniques, as well as methods based on solution titration, i.e., the Fratini test and its simplification, also referred to as the saturated lime (SL) [78] test or lime consumption (LC) test [28]. Among the indirect methods, the authors [78] include those that allow the determination of the pozzolanic activity of the material on the basis of the study of the characteristic which is affected by this activity. These features can be, e.g., the compressive strength of mortar specimens, the electrical conductivity of a saturated solution of Ca(OH)_2_ in which the tested material is placed [80,81,82,83], or the determination of the amount of heat released in calorimetric tests [84,85]. 

A wide review of methods of testing the pozzolanic activity is presented in paper of Tkaczewska [79]. The methods described therein were divided into chemical and physical. Although some of them come from currently withdrawn standards, it is worth quoting them here to show the richness and diversity of approaches to this topic, which even with this review will not be exhausted.

One of the methods is the measurement of portlandite consumption by SCM using thermal analysis methods, i.e., TGA in combination with DTA [86]. In this method, the measure of pozzolanicity is the amount of calcium hydroxide bound by the tested material. It is defined as the difference between the initial Ca(OH)_2_ mass and the remaining unbound mass. The latter is determined in a thermal test from the loss in mass of the sample in the temperature range 490–510 °C, which corresponds to the decomposition of portlandite. This method has several weaknesses which must be taken into account when using it. First of all, the temperature of calcium hydroxide decomposition may vary depending on the alkali content or even grain size [87]. Another factor is the possibility of loss of portlandite in the sample due to carbonation and mass loss of other hydration products in the temperature range corresponding to calcium hydroxide. These problems were identified by Kim and Olek [88] and they proposed an appropriate test procedure.

TGA/DTA testing requires expensive equipment, so it is not a method that can be used in many laboratories that are less well equipped. To address such situations, Mendoza and Tobón [89] have carried out a comparative study of the results of weight loss measurement of calcium hydroxide mixture with potentially pozzolanic material using a moisture analyser. They succeeded in demonstrating that if a sufficiently high sample heating temperature in the moisture analyser is ensured (in the quoted article it was 230 °C), the mass loss at this temperature obtained using both methods is characterised by a high correlation (R^2^ = 0.971), while the mass loss in the moisture analyser is slightly higher. With appropriate assumptions, it is possible to test the pozzolanic activity of the materials by determining the amount of water lost by the hydration products, which increases with the progress of the pozzolanic reaction. 

Fratini is identified with at least two different methods to test the activity of pozzolan. The first one [90,91,92] consists in the determination of the amount of calcium ions bound by the tested material mixed with cement. A sample of the ground test material is placed in a Ca(OH)_2_ suspension at 40 °C for eight days [93]. During this time, the sample is shaken from time to time. The suspension is then filtrated and the total alkalinity of the filtrate is determined by titration with 0.1 mol HCl solution. The next step is to neutralise the filtrate with ammonia and to determine the amount of CaO in it by complexometric method. The concentration of OH^−^ ions (total alkalinity) and Ca^2+^ ions determined in the test is compared with the calcium hydroxide solubility isotherm curve. The location of the result below the curve means that the material is active pozzolan, the greater its activity, the greater the distance of the result beneath the curve.

The second Fratini method is the strength method. It consists in testing the strength of two series of mortar samples prepared with the use of cement and the tested material in the amount of 30–50% of the hot water. One series of samples is stored for seven days in water at a temperature of 20 °C, and the other only three days, after which it is transferred to water at 50 °C for another four days. The difference in strength of both series of samples proves pozzolanic activity of the tested material [94]. 

The method according to the PN-EN 450-1:2012 standard [95] is based on a similar methodology as the Fratini strength method. It is designed to test the activity of fly ashes, however, it can also be applied to other materials showing the pozzolanic activity. It is based on the determination of the pozzolanic activity index (IAP), defined as the ratio, expressed as a percentage, of the compressive strength of a mortar with a fixed proportion of binder, water and sand of a specific grain size made in two versions. In the first version, 25% of cement is replaced by fly ash (or other pozzolanic material), while in the second version, 100% cement is used. The standard requires that it is Portland cement CEM I 42.5R. Pozzolanic activity index is calculated on the basis of the strength test after 28 and 90 days. After 28 days it should reach ≥75% and after 90 days it should be ≥85% for the test material to be considered as active pozzolan. The method of measuring the pozzolanic activity index gives results that closely correlate with the determination of lime concentration in saturated solution with the test material [78,80,81,96]. This method is often referred to in the literature as SAI (strength activity index) and varies, depending on the standard on which it is based, in the composition of the tested mortar (the amount of cement exchanged for the tested material, e.g., 20% [97] or 30% [98] instead of 25%) and the required minimum SAI value (e.g., it is 80% after 28 days [98] or 75% after seven and 28 days [97]).

Apart from testing the strength of mortars containing cement, there are also methods based on testing the strength of mortars prepared from slaked lime and the tested material. One such method is contained in the Serbian standard SRPS B.C1.018:2015 [99]. A mixture of slaked lime, pozzolana, sand and water with a mass ratio of 1:2:9:1.8 is prepared for testing. From this mixture samples of 40 × 40 × 160 mm are formed, which are stored for 24 h in temperature 20 ± 2 °C with a relative humidity of 90%. Then they are placed in thermostat chamber at temperature 55 ± 2 °C for five days. Before testing, specimens are stored again 24 h in temperature 20 ± 2 °C and relative humidity of at least 90%. Compression and flexural strength of the specimens is the basis for the division into three classes of pozzolanic activity. The requirements for each class are as follows: class I—bending strength ≥ 4.0 MPa, compressive strength ≥ 15.0 MPa; class II—bending strength ≥ 3.0 MPa, compressive strength ≥ 10.0 MPa; class III—bending strength ≥ 2.0 MPa, compressive strength ≥ 5.0 MPa.

The method based on the study of the influence of pozzolanic material on the strength of cement mortar is also Graff’s method [79]. In this method three series of mortars differing in binder are prepared. In the first series it is Portland cement and in the second series it is a mixture of Portland cement and the tested material, with the content of the latter in the samples: 15, 30, 50, 70 and 90%. The third series is prepared just like the second series, but replacing the test material in the binder with quartz sand in the same proportions. After seven, 28 and 90 days from the preparation the sample is subjected to strength tests according to PN-EN 196-1:2006 [100] and the pozzolanity number is calculated according to Equation (2):

P = (a − c)/(b − c),(2)

in which one: P is the pozzolanity number, a is the compressive strength after 28 days of the second series of samples, b is the compressive strength after 28 days of the first series of samples and c is the compressive strength after 28 days of the third series of specimens.

The method contained in the withdrawn Russian standard GOST 6269-54 [101] is similar to the first of the Fratini methods described above. According to it, the measure of pozzolanic activity is the total amount of calcium hydroxide bound by pozzolana during 30 days. A sample of the investigated pozzolana (2 g) is placed in a container with 100 mL of saturated calcium hydroxide solution, which is sealed and occasionally shaken vigorously. After two days, 50 mL of the solution is taken and is titrated with HCl in the presence of methyl orange. Then 50 mL of saturated calcium hydroxide solution is poured into the cylinder. This procedure is repeated every two days and the whole test takes 30 days. A modification of this method is using barium hydroxide instead of calcium hydroxide and determining its loss in time [79]. Barium hydroxide dissolves better in water but simultaneously carbonates faster [102]. 

Chemical methods include the method contained in the withdrawn ASTM C379-65T [103]. It assumes the determination of the pozzolanic activity of the material on the basis of the determination of the amounts of soluble SiO_2_ and Al_2_O_3_ in it. The test material should be placed for 1.5 h in a 1-molar solution of sodium hydroxide at 80 °C. The total content of dissolved SiO_2_ and Al_2_O_3_ above 20% indicates that the material shows pozzolanic activity. The same standard also includes a method based on testing the strength of the lime - pozzolanic mortar. Samples of such mortar should be stored for seven days at 65 °C in a humid atmosphere, then cooled to 23 °C and kept at this temperature for another 21 days, also in a humid atmosphere. The material exhibits adequate pozzolanic activity if the samples reach compressive strength ≥4.1 MPa. ASTM C379-65T was withdrawn in 1966 and replaced by ASTM C593-95 [104], which only contains the latter method.

Chapelle test consists in boiling the pozzolana sample in Ca(OH)_2_ solution for 16 h and then determining the calcium hydroxide content in the filtrate [105]. A measure of the pozzolanic activity of the material is the difference in Ca(OH)_2_ content in the initial solution and in the filtrate. Nino et al. [106] have modified this method for their research. They used 300 mL of saturated calcium hydroxide solution at 20 °C, when the solubility of this compound in water is 1.65g/L. They placed 0.5 g of kaolin, almost as much as Ca(OH)_2_ in the solution. After heating the solution to 100 °C, part of the calcium hydroxide was precipitated, but during the test, as part of Ca(OH)_2_ reacted with kaolin, the precipitate dissolved, keeping the solution saturated. The test was extended to 24 h and the solution was mixed at 150 rpm.

Another chemical method is the Feret–Florentin method, about which Feret mentioned in [107] and which he credited to Florentin [108]. The ground sample of test material is shaken for 10 min in 100 mL of HCl (30%) and then the amounts of SiO_2_, Al_2_O_3_ and Fe_2_O_3_ that have entered the solution are determined. Then a further aliquot of the test material is mixed 1:1 with Ca(OH)_2_ adding enough water to give the prepared mixture a plastic consistency. The prepared mixture is stored for three days in a humid atmosphere and then it is placed in water at 15 °C. After one, four and 26 weeks from the preparation of the mixture, part of the mixture is taken, dried, ground and the SiO_2_, Al_2_O_3_ and Fe_2_O_3_ content is determined in the same way as in the case of the test sample itself (shaking in 30% HCl). Pozzolan activity is measured by the increase in the amount of leached oxides in a sample of test material with Ca(OH)_2_ compared to a reference sample containing the test material itself. 

Feret himself is the author of the method in which pozzolanic activity is tested firstly by treating the material with a 30% solution of HCl and then with a 25% solution of NaOH. The measure of pozzolanicity is the amount of material tested, expressed as a percentage, which has migrated to the solution. In order to consider that the material has a high pozzolanic activity, at least 60% of its mass should be dissolved. The simplification of the Feret method is the Jarrige and Decreux method [109]. It consists in treating the tested material with HCl for five and 30 min. The measure of pozzolanic activity is an increase in the amount of dissolved material with an increase in the leaching time. Material characterized by good pozzolanic activity should have a difference in solubility of more than 10%.

The Guillaume method [110] consists in comparing the content of insoluble parts of two blended cements, one of which contains 20% by weight of the tested material and the other 20% by weight of quartz sand. Both cements are treated with HCl and the amount of insoluble residue is then determined. In the next step the cements samples are heated at 1000 °C for 1 h, after which they are treated with HCl as previously unheated samples. The pozzolan activity is determined by the increase in solubility of the cements after heating. If active SiO_2_ is present in the test material, it reacts with calcium oxide or alite to form HCl-soluble calcium silicates. Cement with added quartz sand does not change significantly its solubility in HCl.

The method proposed by Battaglino and Schipp [111] is based on two criteria: the determination of specific surface area of hardened cement paste prepared with the addition of 30% of tested material and the determination of free Ca(OH)_2_ content. The tests are carried out after different hardening periods. A sample of the paste should be ground and then the 30–60 μm fraction should be separated. The specific surface area is determined by the BET method and the free Ca(OH)_2_ content by the ethylacetylacetate method (Franke method) [112]. A material with good pozzolanic activity is considered as one which reacts quite slowly with calcium hydroxide and leads to a large specific surface area.

Another suggestion for the test of pozzolanic activity is the R^3^ method (rapid, relevant and reliable). A detailed description of it and the underlying research can be found in [113,114]. This method has two variants. The first one is based on a heat release test in an isothermal calorimeter, in which the mixture is placed. It consists of the material tested, calcium hydroxide (additionally calcium carbonate in the case of testing mixtures of LC^3^) and potassium hydroxide as well as potassium sulphate both added in such quantities that the reaction environment has a similar composition to a pore liquid in concrete. Detailed compositions of the mixtures are given in the above mentioned publications. At the method development stage the prepared mixture was tested in a calorimeter at 20 °C and 40 °C for seven days. Since the amount of heat released at the higher of these temperatures after 1 day is equal to the amount released at the lower one after six days, further analyses were based on the results obtained after one day at 40 °C. The second variant of this method starts with the preparation of a mixture with the same composition as the first variant. This mixture is placed in a sealed container and treated at 40 °C for one day. The sample is then dried at 110 °C until its mass change per day becomes less than 0.5%. The sample so dried is exposed to a temperature of 400 °C for two hours and then cooled to 110 °C. The sample is weighed before and after heating at 400 °C and the quantity of water bound in the hydration products is determined from Equation (3):

W_b_ = (m_s_ − m_c_)/m_s_ [%], (3)

In Equation (3) W_b_ is the quantity of water bound by the hydration products, m_s_ is the mass of the sample after drying to a constant mass but before exposure to 400 °C and m_c_ is the mass of the sample after heating and then cooling to 110 °C. According to [113], the results obtained show a high correlation with the results of the strength tests and the Chapelle test. A quick test of pozzolanic activity based on the conductivity test was proposed by Luxán et al. [115], developing an idea presented by Raasek and Bhaskar [116]. The idea of this method is to test the conductivity of a saturated solution of Ca(OH)_2_ kept at a constant temperature of 40 ± 1 °C, in which a sample of the tested material, dried earlier at 105 ± 5 °C, is placed. Pozzolan activity is measured by the difference of the compensated conductivity of the solution at the start of the test and after 120 s. According to the classification proposal of [115], a material is considered as non-pozzolanic if the difference in compensated conductivity is less than 0.4 mS/cm, and if it is greater than 1.2 mS/cm, the material is considered as having good pozzolanicity. Between these two results there are materials which show variable pozzolanicity. In its original form this test was not resistant to interference from soluble salts increasing the conductivity of the solution. Payá et al. [80] proposed several modifications to this test, such as extending its time, carrying it out at different temperatures or using unsaturated calcium hydroxide solution. The most important amendment to the test methodology was the proposal to additionally measure the conductivity of distilled water, in which the material sample was placed. Both curves obtained in this way (conductivity of calcium hydroxide solution and distilled water) should be subtracted from each other and analysed. 

Each method has its strengths and weaknesses, so it is best to use more than one method to obtain as complete a picture as possible of pozzolanic activity of the tested material. Danatello et al. [78] comparing the saturated lime method, Fratini test and SAI concluded that the first method is the most questionable. It did not show any correlation with two other methods, between which a high correlation was found. Therefore, they recommend using the Fratini test together with SAI, and also one of the direct methods. There is also a number of voices that suggest only direct methods should be used in the determination of pozzolanic activity [117]. 

## 4. The Results of Investigations of Clays from Various Worldwide Deposits

Maier et al. [10] examined 11 clays from various deposits in Germany. The calcination temperature of each clay was determined from the DTG results by adding 100 °C to the main peak temperature of the dehydroxylation process. The mineralogical composition of the raw materials and the proportion of amorphous phase after the calcination process were determined. Reactivity was investigated using R^3^ calorimetry according to [114] (described in the previous section) and solubility of Al and Si ions. The results showed a high agreement between the results of the last two tests. The authors also came to a conclusion that in order to properly determine the suitability of clay as a raw material for SCM production it is not enough to know the chemical composition, especially in case of clays with low kaolinite content. In those cases other minerals play an important role, therefore, it is necessary to determine the complete mineralogical composition. The R^3^ calorimetric test can also be useful in the evaluation of this type of clays.

Tironi et al. [11,118] have studied five kaolinitic clays from various deposits located in Argentina with kaolinite content between 16% and 94%. These clays were tested by X-ray diffraction (XRD) and Fourier transformed infrared spectrometers (FTIR) before activation. These tests determined the phase composition of the clays and the order or disorder of the kaolinite they contain. The latter determination was performed in accordance with the methodology proposed by Bich [56], which consists in calculating the P_0_ coefficient, which is the quotient of the intensity bands at 3620 cm^−1^ and 3700 cm^−1^ obtained in the FTIR test. If P_0_ > 1, then according to [56], kaolin has well-organised structure and P_0_ < 1 indicates disordered structure. In the case of three clays with intermediate kaolinite content the structure of this mineral was disordered. The clays were heated to 700 °C where they stayed for 5 min (and the whole heating process took 1 h). An XRD study after calcination showed that the time and temperature were sufficient to transform the kaolinite into metakaolinite. After calcination, pozzolanic activity was also determined by the Fratini test and the electrical conductivity test (previously described). The compressive strength of mortars in which 30% of cement was replaced with ground calcined clay was also tested. On the basis of the results of the latter test, a model was proposed in which the strength of the mortars (after seven, 28 and 90 days), in which 30% OPC was replaced with ground calcined clay, depends on the content of kaolinite in the clay, on the specific surface defined by the Blaine method and on the inverse of the P_0_ coefficient. The authors proved that the results obtained from the application of the proposed model are highly consistent with the test results. Thus, they showed that the calcined clays’ pozzolanic activity depends not only on kaolinite content but also on the degree of order of its structure and specific surface of the clays after calcination and grinding.

Huenger et al. [119] selected three clays from Lower Lusatia deposits and tested their pozzolanic activity after burning. Among the selected clays, one was characterized by a high content of quartz (about 60%) co-occurring with smaller amounts of kaolinite and illite, the second one consisted mostly of kaolinite (about 60%) and quartz and a small amount of illite, and in the third one more than half of the composition consisted of quartz, and the other clay minerals mentioned earlier were present in smaller, similar amounts. Each clay was calcined separately, as well as their mixtures. Three temperatures of heat treatment were applied: 600 °C, 650 °C and 700 °C. The study of mineralogical composition of the calcined clays showed a slight decrease in the illite content and a significant decrease in kaolinite content accompanied by a proportional increase in the amorphous phase. The studies of pozzolanic activity allowed to formulate a conclusion that the mixture of kaolinite-rich clay with quartz-rich clay in the proportion 60:40 allowed to obtain a material with satisfactory parameters.

Beuntner and Thienel [120,121] presented the results of research on clays with clay content of about 70% (with a predominance of illite) from deposits located in southern Germany. In [120] the authors compared the material obtained by calcination of clay with kaolinite content of 25% in two technological processes. One was carried out on a laboratory scale and the other on an industrial scale. The comparison of research results showed that calcination in industrial conditions allowed to obtain material with slightly lower pozzolanic activity, but still at a satisfactory level. It was also shown that the mineralogical composition of clay subjected to heat treatment varies according to grading. Whereas [121] was devoted to the study of selected technological, mechanical and durability properties of concrete prepared from nine different types of cement with different exchange rate of the cement with calcined clay, which was used as a Type II addition. Mixtures of clay with cement in most cases showed slower development of strength in the initial period. However, later on, the strength increase accelerated and in the case of mixes with 20–25% clay, the strength exceeded the values obtained for concrete with sole cement. Moreover, it was found that the replacement of cement parts with calcined clay reduces consistency, bleeding and shrinkage.

Papers [122,123,124] are devoted to research on the possibility of using kaolinite waste fraction from Amazonian deposits for the production of calcined clays, which were an intermediate product for further zeolite production. The research was carried out on waste fractions of kaolin from deposits of the Brazilian Amazon calcined at various temperatures in the range 550–800 °C for 2 h. Results of the tests showed that the wastes were composed primarily of coarse-grained kaolinite, which can be an excellent starting material for pozzolana and zeolite production. 

Akasha [125] presented the results of research on the use of clay obtained from five locations in Libya and calcined as a partial substitute for cement. The studied clays contained from 30% to 90% of kaolinite and, besides that, mainly quartz (and in one case additionally a small amount of illite). The clays were calcined at 800 °C for two hours. Subsequently blended cements were prepared in which the share of calcined clays was 10%, 15% and 20%. The obtained results do not show a clear correlation between pozzolanic activity and kaolinite content in the raw material. In comparison with the control mortar, in which pure cement was used, only the mortar containing calcined clay containing initially 30% kaolinite (the least) and 70% quartz showed higher strength at all test dates and at all exchange rates. 

Chakchouk et al. [126] have selected six clays from five Tunisian deposits located in different parts of the country. Samples of clays have been tested for basic properties such as plastic limit, liquid limit and plasticity index. Blue methylene test and calcimetry were also carried out on them. The chemical composition and qualitative mineralogical composition with XRD was determined. The chemical composition was used to verify the criterion of pozzolan activity according to two standards: ASTM C618 [97] and an unspecified Indian standard. The results showed that with the exception of one of the clays, which did not meet the Indian standard of CaO < 10%, the others should be a good material for the production of pozzolanic active materials. These five clays were subjected to calcination at 600 °C, 700 °C or 800 °C, and then their pozzolan activity was determined by examining the compressive strength of clay mixed with calcium hydroxide in two proportions CC:CH = 1 and CC:CH = 3. The highest strength was obtained in case of two clays with the highest content of kaolinite and at three times the mass prevalence of calcined clay to calcium hydroxide. Their optimal calcination temperatures are 700 °C and 800 °C. In the case of two of the remaining clays, the measured pozzolanic activity was moderate and the optimal temperature (among the applied ones) was 800 °C. The fifth of the examined clays showed weak pozzolanic activity, which the authors attribute to high quartz content.

The reported results of the research indicate that it is necessary and advisable to develop a research protocol which could be used as a standard for examining clays from unexplored local deposits. The diversity of clays from different deposits in the same country indicates the need to take into account the specific mineral composition of a particular clay in developing and optimising its calcination technology.

## 5. Calcined Clay in Cement and Concrete Technology

The issue of calcined clays as SCM cannot be considered in isolation from the technological determinants related to the use of this active pozzolanic material in the production of blended cements and concrete. In [41] Samet et al. applied the response surface methodology (RSM) [127,128] to determine the value of: calcination temperature, specific surface of calcined clay and its share in the blended cement to obtain optimal parameters of the blended cement. Normal consistency, setting time, stability of expansion, mechanical properties and the specific surface of the calcined clay were taken into account. The presented methodology is universal and can be used to determine the optimal composition of cement blends with different types of calcined clay.

The issue of optimization of the composition of cement blends with calcined clays was also considered by Pierkes et al. [129]. Due to a large number of variable parameters, they planned their research using the DOE (design of experiments) with statistic tool MINITAB^®^. Four types of Portland clinker and three types of clays (illitic, kaolinitic and chloritic) were used in the research. The clay was added to the clinker in amounts of 20% and 40%. Additionally, the anhydrite was added in a different amount (2% or 4%). The research included compressive strength after two, seven and 28 days and studies of hydration products after 3 h and two days. The authors came to the conclusion that there is space to improve the strength parameters of calcined clay cements by selecting both the appropriate chemical and mineralogical composition of the raw materials and adjusting the amount of sulphates accordingly.

Siddique and Klaus [130] have reviewed widely the literature on the influence of metakaolin on the properties of mortars and concrete. The analysis included fresh properties, properties of hardened materials and durability properties of concrete. They stated, that the use of metakaolin had a positive impact on both early age and long-term strength properties of cement paste, mortar cement and concrete. It also reduced water absorption of concrete by capillary suction (i.e., its sorptivity [131]), as well as other parameters that may reduce the durability of the material (e.g., permeability) due to the refinement of pore structure. Moreover, mortars and concrete performed with metakaolin replacing cement in the amount of 10% and 15% showed very good chemical resistance, including sulphate corrosion.

Paiva et al. [132] have studied properties of fresh and hardened concrete prepared with metakaolin with the pre-set workability. The influence of metakaolin particles dispersion achieved with water and polycarboxylic acid based HRWRA on concrete structure was researched and discussed. The results clearly favoured the use of HRWRA to control the workability of mixture with such fine additions as metakaolin. The use of water alone had resulted in increased porosity and the formation of agglomerates of calcined kaolin, which has had a negative impact on the strength parameters of the hardened material.

Research on the compatibility of superplasticisers with cement mixes containing metakaolin and limestone was undertaken by Zaribaf et al. [133]. They used cement type IL according to ASTM C595 [134], containing limestone and commercially available metakaolin. Four admixtures each with different chemical base were used as superplasticisers. The results, which were discussed, included flowability measured by the minislump size, compressive strength, hydration heat measured in isothermal calorimeter and setting time. On the basis of the results obtained, the authors came to the conclusion that admixtures based on polycarboxylate ether (PCE) and polymelamine sulfonate (PMS) were the most compatible.

An extended analysis of the efficiency of superplasticizers depending on the mineralogical composition of calcined clay and the type of cement (OPC or PLC) was conducted by Sposito et al. [135]. Apart from the two types of cement mentioned above, they used four types of calcined clay in their research, one of which was a typical clay with different mineralogical composition, one was commercially available metakaolin and the other two contained in its raw form more than 90% of one of the minerals: illite or muscovite. Quartz powder was also used to evaluate its impact on the rheology. Three different admixtures were used as superplasticisers. The tests were carried out on both clay and cement mixes as well as on clinker-free suspensions. The results and their analysis allowed the conclusion that zeta potential, total surface area and water demand were reliable predictors of superplasticizer demand with the exception of mixtures with calcined muscovite. However, the latter reservation should be alleviated in the case of clays with different mineralogical composition, in which muscovite content is a medium. The share of quartz powder in the tested systems for the variety significantly improved workability.

Nied et al. [136] investigated the synergistic effect of the use of metakaolin and limestone on the mechanical properties and phase assemblage of the blends composed in this way. For this purpose they prepared 12 blends with a fixed OPC content of 60% and a variable content (10%, 20%, 30% and 40%) of metakaolin of two qualities, limestone (10–40%) and quartz instead of limestone (20%) in reference blends. The authors concluded that part of metakaolin in binary blends with OPC could be replaced with limestone, which positively affects both workability and 28-day compression strength, with the exchange ratio depending on the quality of metakaolin. Moreover, it was observed that the addition of limestone stabilises ettringite, refines pore structure and alters the hydration of OPC/metakaolin blend at early age. 

Beuntner et al. [137] presented the results of a study on the possibility of using calcined clay in high-performance concrete. It is worth emphasizing that they used not only metakaolin, but also mixed layer clays, which on the one hand occur in much larger quantities in world clay deposits, but on the other hand are treated as “poorer sisters” of kaolinite. The results of the research, which included, among other things, compressive strength, modulus of elasticity and porosity, showed that calcined clays, including those obtained from raw mixed layer ones, can successfully replace silica fume in HPC, although this may require doses of superplasticisers larger than those currently recommended by their manufacturers.

The use of calcined clays as binder components can be combined with the simultaneous use of other types of pozzolan. Mechti et al. [138] presented results of a study in which finely ground sand was used as an additional component which, in spite of its crystalline structure, showed the pozzolan activity. The aim of the study was to determine the optimal proportions of cement blend components and, as additional parameters, fineness of ground sand as well as temperature of calcination of clay. The plan of the experiment included 28 mixtures characterised by different values of the optimised parameters. The analysis of the results showed that the optimum composition under the assumed assumptions was the one containing 80% of cement, 15% of sand with fineness lower than 40 μm and 5% of clay calcined at temperature 750 °C.

Two different pozzolana (calcined clay and fly ash) in cement mixes were also used by Ng and Østnor [139]. This combination had its justification in the different characteristics of both pozzolana materials. Fly ash is a material which very slowly increases the strength of cement-based composites, while calcined clays, especially when used with limestone, are significantly faster in the process of pozzolan reaction. In the presented studies, 20% of OPC was exchanged for fly ash, clay or mixtures of these two pozzolana in different proportions. The results showed that the simultaneous application of fly ash and calcined clay improved the workability of the mixture, which is usually impaired when using only calcined clays as a substitute for cement. It has also been shown that the combination of these two pozzolanic materials allows strength values to be achieved after 28 days at the level of mixtures using only OPC.

Workability and hydration of blended cements with calcined illitic clay have been studied by Marchetti et al. [140]. They used two types of ground, calcined illitic clays and OPC, the content of which was reduced in favour of each clay by 15%, 25% and 35% by weight. Cement mixtures were tested for their packing density (according to [141]), heat release, flow, compressive strength and some additional parameters related to the hydration course. The obtained results allowed to state that with the increase of the share of calcined illitic clay in the mixture, its workability decreased. As far as heat release is concerned, the clay caused a decrease in the amount of heat generated during the first 48 h, but at the same time it did not cause any significant delay in the hydration process. 

Machner et al. [142] have studied the possibility of using dolomite to replace limestone as a third component in mixtures with OPC and metakaolin. The test mixtures were composed of Portland clinker, dolomite, limestone, metakaolin and gypsum. A total of six blends were prepared, which together with the cement gave seven different series. In all mixtures the ratio of OPC to metakaolin was constant at 6:1 and the addition of limestone or dolomite was 5%, 10% or 20%. The results showed that if the 90-day strength was taken as a determinant, there was no significant difference between limestone and dolomite. The latter mineral, however, showed less reactivity, which was particularly evident at low curing temperature.

As it can be seen that the addition of calcined clay reduces the early strength of cement composites due to lower reactivity of pozzolana. Paper [143] presents the results of research on the possibility of acceleration of cement strength development in the initial period by using CSH seeds. The clay obtained from a deposit in southern Germany, which after calcination was mixed with OPC in the proportion 20:80, was used in the study. Thus, the prepared cement, corresponding to type CEM II-A/Q, was enriched with 1%, 2% and 3% of CSH seeds admixture. In another series 5%, 10% and 20% of the cement mass was replaced with the microlimestone. There were performed calorimetric tests, TGA and SEM investigations as well as flow and compressive strength tests on mortars. The results allowed to recommend the addition of 3% of CSH seeds admixture or 10% microlimestone to optimize the early strength development of cement with 20% addition of calcined clay.

There is a consensus among researchers that the replacement of part of cement with metakaolin (up to 15%) reduces drying shrinkage of concrete compared to material without metakaolin or with silica fume [144,145,146]. However, as far as autogenous shrinkage is concerned, the results are divergent. Gleize et al. [147] investigated the influence of metakaolin on the autogenous shrinkage of cement paste. They used CEM I 52.5 cement, which was replaced by metakaolin at 0%, 5%, 10%, 15% and 20% rates. Mixtures were prepared with two water to binder ratios: 0.5 and 0.3. In the latter case a superplasticizer was used. The results allowed to formulate a conclusion that long-term autogenous shrinkage decreased with increasing proportion of metakaolin in the binder. The majority of this effect was caused by the pozzolanic activity of the applied metakaolin, and not by the effect of dilution of the cement with the addition.

A review of the studies on the use of calcined clays in cement and concrete technology shows an overwhelming predominance of articles devoted to various aspects of application of metakaolin. It is the best-researched calcined clay mineral, although even in its case the results obtained are far from being generalised. Although kaolin is a grateful object both for testing and for subsequent use after calcination, due to its limited availability, more extensive research into the properties and application of clays with different mineralogical compositions is necessary. 

In addition, many of the work presented is based on results obtained using pastes or mortars cement. Concrete testing results are in a clear minority. This is probably due to the need to produce larger amounts of calcined clay with homogeneous parameters, which can be difficult in laboratory conditions. Nevertheless, this is an important direction of research that deserves more attention.

## 6. Lime Calcined Clay Cement (LC^3^)

The cement consisting of Portland clinker, calcined clay (preferably rich in kaolinite), calcium carbonate and gypsum is described in literature as LC^3^ [148]. It is a solution for a demand for cement that is more environmentally friendly, the production of which takes place with lower CO_2_ emissions to the atmosphere, and which at the same time is not inferior to ordinary Portland cement with a clinker content of at least 90%. The abundance of raw clays and limestone is also an important factor in the development of LC^3^ production, in contrast to the shrinking resources of good quality fly ash or even their unavailability in some countries.

The most common composition for the LC^3^ is 50% of ground Portland clinker, 30% of ground calcined clay, 15% of ground limestone and 5% of ground gypsum. Such a mixture is sometimes referred to in the literature as LC^3^-50. Other proportions of components are also possible [149], but it is known that cement prepared according to the above composition, according to the research [113,150], reaches mechanical parameters corresponding to OPC already after seven days of hydration, provided that the clay contains at least 40% kaolin. 

An important aspect of LC^3^ production is its cost and profitability. Papers [151,152] present the results of economic analysis of production of this type of ternary blend in India. The authors concluded that the production of LC^3^ is economically viable if the following conditions are met: the cost of fly ash will be high, the quality of fly ash will be low, the acquisition of fly ash will require longer transport than the acquisition of clay and the quality of the extracted clay will reduce the amount of clinker in cement. Most of these conditions are already met, for example, in countries which do not have sources of good quality fly ash. Given the restrictions on the use of fossil fuels in energy production, these conditions may soon be met automatically. 

Apart from the economic aspect, the durability and performance of concrete with LC^3^ is no less important. Several publications have been devoted to this issue. In papers [153,154] the authors assessed the pore structure in concrete made of three types of cement: OPC, Portland pozzolana cement with 30% of Class F fly ash and LC^3^. The results of the study showed a much finer pore structure in concrete with LC^3^, which was proved in the mercury intrusion porosimetry study. Additionally, the conductivity of the concrete obtained in this way turned out to be lower, which allows one to assume that it will have higher resistance to the penetration of harmful ions into its structure. In short, its durability can be predicted to be significantly higher than that of other concrete series compared in the work.

Khan et al. [155] discussed carbonation of LC^3^. The study covered investigation of concrete with cement, in which 15%, 30% and 45% of the mass was replaced by a mixture of calcined clay (containing about 50% metakaolin and 50% quartz) and limestone in a ratio of 2:1. For comparison, two series of concrete with OPC were used. One of them had the same aggregate, water and binder ratio as the LC^3^ concrete series, and the other modified proportions and quantities. The results indicate that concrete with LC^3^ and 15% cement exchange rate showed higher carbonation resistance than concrete with OPC. At 30% a slight advantage was gained by concrete with OPC, and at a 45% cement exchange rate to a mixture of calcined clay and limestone, concrete with LC^3^ showed a high carbonation rate. The same authors [156] presented the results of the same concretes according to their fresh properties, strength, porosity and drying shrinkage. They showed that the concrete with LC^3^ was characterized by worse workability. As far as strength was concerned, the highest was reached by concrete with a 15% exchange rate of cement, which also had the lowest porosity. As far as drying shrinkage is concerned, all series of LC^3^ concrete were lower than OPC concrete. 

An interesting approach to the issue of carbonation is presented in paper of Joseph et al. [157]. The authors have been tempted to create the protocol of testing the durability of LC^3^ (although not only) with respect to carbonation. This protocol included the determination of indicators of the carbonation process, such as diffusivity of CO_2_, hydration and carbonation products, total possible carbonation, and pH change due to the carbonation and rate of carbonation. The paper also contains a suggestion to determine the above mentioned elements and the possibility to apply the presented approach to other issues concerning concrete durability.

Nguyen and Castel [158] evaluated the resistance of concrete with LC^3^ to chloride diffusion. Three series of concrete were made for research purposes. One contained only general purpose cement corresponding to Type I cement according to ASTM C150/C150M [159]. In the remaining two 15% and 20% of the cement was replaced by a mixture of calcined clay and limestone in the ratio 2:1. The results indicate that the use of LC^3^ in concrete significantly increased its resistance to chloride diffusion. The continuation of the research on chloride diffusion resistance Nguyen et al. presented in [160], where they compared concrete made with OPC with concrete containing calcined clay obtained in two technological processes: flash calcination and calcination in rotary kiln. Due to differences in activity of both types of calcined clay, the authors decided to apply different proportions of general purpose cement (as per Australian Standard AS 3972 [161]) to calcined clay (20% for flash calcined clay and 44% for clay calcined in rotary kiln). This variation was due to the intention to obtain the appropriate compressive strength of the hardened concrete (>45 MPa). The results indicated four times higher chloride diffusion resistance of concrete with LC^3^. The difference between the results of concrete made with different types of calcined clay turned out to be insignificant, which led the authors to conclude that it is crucial in this case to properly select the proportion of cement exchanged for calcined clay to meet the requirements of specific strength of concrete exposed to chloride ingressives. 

A different approach to chloride diffusion research was applied by Yang et al. [162]; they discussed the results of simulations of this phenomenon in LC^3^ concrete and concrete with fly ash compared to it. The authors conducted extensive simulations taking into account many elements affecting the course of chloride diffusion in hardened concrete. The results showed that both concretes had comparable service life even though the LC^3^ concrete contained less clinker. The conclusions also indicated important parameters that should be introduced to the simulation in order for the results to be reliable.

Nguyen and Castel [163] presented the results of research on corrosion of reinforcing steel in concrete made using LC^3^. This issue is important because of the lower alkalinity of this type of concrete due to the lower amount of portlandite, which is consumed by calcined clay in the pozzolanic reaction. The research, carried out over 500 days, allowed the conclusion to be drawn that performance of reinforced concrete prepared with LC^3^ was comparable to that for concrete with OPC in the propagation phase. 

Rengaraju et al. [164] also investigated corrosion of steel in concrete with LC^3^. They presented the results of corrosion testing of steel in three concrete mixes prepared with the use of: OPC, a blend of 70% OPC with 30% of fly ash and a blend of 50% OPC with a 50% mixture of limestone and calcined clay. In their previous work [165] they carried out tests according to AASHTO T 358 standard [166], which showed that concrete with LC^3^ showed very high resistivity, which is a good predictor of resistance to chloride ion penetration and, consequently, to corrosion of reinforcing steel. As a continuation of the tests, corrosion tests of steel in concrete were carried out using the method included in ASTM G109 [167] and the impressed current corrosion test method. In the conclusions, apart from the statement that LC^3^ cement composites showed higher resistance to chloride ingress and better protection of steel against corrosion, the authors also claimed that the corrosion products of steel in concrete with LC^3^ were less expansive and thus less destructive to concrete.

The study of corrosion of reinforcement in concrete made with LC^3^ was also conducted by Pillai et al. [168]. They determined such concrete parameters as chloride diffusion coefficient, ageing coefficient and chloride threshold for seven mixtures containing OPC and blends of OPC with pulverised fuel ash (PFA) or with calcined clay and limestone (i.e., LC^3^). Using these parameters they determined the service life of the two construction elements. The results showed that a construction made of LC^3^ or blended cement with PFA will had a significantly longer service life with a much smaller carbon footprint.

The issue of carbon footprint is focusing our attention on the eco-efficiency of LC^3^. In [169] the authors have undertaken the assessment of the sustainability of LC^3^ using two methods: life cycle analysis and eco-efficiency. The first method was used to assess the environmental impact of LC^3^ production compared to OPC and Portland pozzolana cement (with 20% zeolite content). The second method assessed how the use of LC^3^ in the construction of a model residential building will increase its eco-efficiency. The calculations led to the conclusion that the use of LC^3^ may lead to a reduction of cement production costs by 4–40% and CO_2_ emissions by 15–30%. 

No less important are the technological aspects related to LC^3^ production. The authors of paper [170] undertook the assessment of suitability of four clays from deposits located in Cuba for LC^3^ production. The results turned out to be promising as they indicated that all examined clays had the potential to be used in ternary blend cement production. Additionally, the authors showed that the pozzolan activity was directly proportional to kaolinite equivalent (KEQ), and that the specific surface area of the obtained cements depended mainly on the calcination temperature of the clays and their mineralogical composition.

Nair et al. [171] presented the results of rheological properties test of cement paste and mortar prepared using LC^3^ in comparison with OPC and Portland-fly ash cement (containing 30% Class F fly ash). The effectiveness of five superplasticising admixtures based on polycarboxylic ethers (PCE) differing in chemical composition and one admixture based on sulphonated naphthalene formaldehyde (SNF) were also compared. The studies carried out have led to the conclusion that larger quantities of superplasticisers were required for blends with LC^3^, with PCE providing a lower viscosity than SNF and the latter additive had to be dosed in larger quantities to not allow to reduce the w/b ratio below 0.4. 

Li, X. and Scrivener, K.L [172] devoted their paper to the comparison of three methods of determination of reacted metakaolin in LC^3^. The authors compared: mass balance [173], thermodynamic modelling with Gibbs Free Energy Minimization Software [174] and the Partial or not Known Crystalline Structure method (PONKCS) [175]. Of the methods analysed, the mass balance approach yielded the most reliable results, although the use of GEMS software was, on the other hand, less time-consuming and required less labour-intensive input. The PONKCS method proved to be reliable in the case of LC^3^-50 mixtures containing clays with metakaolin content above 60%. 

Papers [176,177] present the results of two approaches to the pilot production of LC^3^ in India. The composition of blends in both cases was almost the same: 50% Portland clinker, 30% or 31% calcined clay, 15% crushed limestone and 4% or 5% gypsum. In the first approach [176] several problems of technological nature emerged, such as too little fineness of the cement due to the use of ground limestone and calcination of only part of the used clay. This resulted in a large scattering of results and lower strength values of LC^3^ concretes. In the second approach [177], the authors did not report any technological complications concluding that it is possible to produce good quality LC^3^ even without the need to change technologies in existing cement plants. 

The influence of the degree of fineness of LC^3^ components on its selected physical and mechanical parameters was examined and presented in [178]. It was assumed that each of the three binder components (Portland cement—55%, calcined clay—30% and limestone—15%) can be ground to two degrees: coarse and fine. In addition, in part of the series, clay or limestone was replaced by finely ground quartz. The results indicate that the greatest influence on the strength parameters of concrete with LC^3^ had the degree of clinker fineness, and a slightly lesser calcined clay fineness. Limestone fineness was important only in the initial period of concrete strength development (up to three days).

Examples of practical application of LC^3^ were presented by Maity et al. in [179]. Four different LC^3^ blends with two types of clay and two types of limestone and, for comparison, Portland Pozzolan Cement were used to produce concrete and various structural elements. All these products have undergone quality control and have been incorporated into the construction of residential buildings. The obtained results showed that the mechanical parameters of LC^3^ concrete can be even higher than those of PPC concrete. The resulting buildings are tangible proof that LC^3^ had moved from the concept and research phase to the first practical application tests.

Dhandapani and Santhanam [180] analysed the impact of the mutual ratio of limestone and calcined clay on the binding and strength development of LC^3^ and blends in which the calcined clay was replaced by Class F fly ash. Binary blends without added limestone were used as a reference. The research showed a clear influence of limestone on the setting of the concrete and much less on the further development of its strength. In addition, concrete using calcined clay achieved a very significantly higher strength after seven and 28 days of hardening.

Ferreiro et al. [181] analysed the influence of the clay calcination temperature in the flash calcination process and the fineness of raw clay on the workability and strength performance of LC^3^. They used two types of clay, which were calcined in two different installations (gas suspension calciner and flash calciner) and at two different temperatures. The clays used consisted mainly of minerals of group 2:1, which was an additional value of the work, as most of the research on LC^3^ was based mainly on clays containing kaolin. The results obtained allowed to conclude that both the calcination temperature and the degree of fragmentation of the material subjected to this process had a significant impact on the workability of fresh LC^3^ mortars and their strength development.

The influence of the method of constituent grinding on rheology and early strength of LC^3^ tested on mortars was examined by Perez et al. [182]. The constituent were ground both separately and jointly. Clinker, limestone and calcined clay ground separately were divided into three fractions, from which LC^3^ was then composed using different combinations of the degree of grinding of the components. In the case of components ground jointly the grinding time varied (25, 45 and 65 min). As far as the latter case is concerned, the results indicate that the longer the grinding time, the higher the strength of the LC^3^ later reached, while the change of mini slump radius over 45 min of grinding time was no longer significant. In the case of separated milling, the degree of grinding of the clinker was crucial and the degree of grinding of limestone was of secondary importance. The influence of the degree of milling calcined clay was not studied by the authors.

The suitability of LC^3^ as a material for 3D printing process has been investigated by Chen et al. [183]. They prepared four mixtures containing different proportions of two calcined clay with lower (49%) and higher (90%) metakaolinite content, and then conducted extrudability and earlier strength tests on them. The results showed that as the amount of metakaolinite in LC^3^ blend increased, its extrudability decreased but at the same time the early strength increased. Therefore, it is crucial to find the optimal content of metakaolinite in LC^3^ used for blends in 3D printing. A referral to the same topic is in [184], in which the authors analyse the influence of viscosity modifying admixture on extrudability of LC^3^ based materials. 

Avet and Scrivener [185,186] focused on the issue of hydration of LC^3^ depending on the content of calcined kaolinite in the blend. The research was carried out on LC^3^-50 blends with different metakaolinite content. The results showed that with calcined kaolinite above 65% hydration of clinker was slowed down after three days due to refinement of the pore structure. However, despite this, the strength was still increasing, which is connected with further reaction of metakaolinite and increase of the C-A-S-H amount.

Zunino and Scrivener [187] dealt with reactivity and mechanical performance of three mixtures containing Portland cement, Portland cement-limestone blend and LC^3^. The distinction of this publication is that the prepared mixtures were cured at a lowered temperature of 10 °C. The same mixtures cured at 20 °C were used as a reference. The research included determination of compressive strength after 1, 7 and 28 days, isothermal calorimetry as well as XRD phase assemblage assessment and MIP pore refinement assessment. They found that LC^3^ cured at lower temperature achieved significantly higher compressive strength than the same cement paste cured at higher temperature. The authors claim that this was an effect of, among others, a slightly different course of hydration of this cement.

In the summary of this chapter, it would be appropriate to repeat the concluding statements of the previous section which indicate the need to extend the interest in clays which may be active after calcination and which do not contain kaolinite or contain small amounts of kaolinite. Another interesting aspect that comes to mind after reading the above article on the practical application of LC3 is the possibility to test the suitability of this type of binder as a base for concrete for road construction.

## 7. Influence of Calcined Clay on the Durability of Concrete

Durability issues are important for each of the cement-based materials used nowadays. A studies related to the durability of cement-based composites prepared with calcined clays are presented in this chapter.

Trümer and Ludwig [188] put forward the thesis that one of the obstacles to the wider use of calcined clays in cement and concrete technology is the lack of information on the long-time behaviour of the concrete. They used clays of various mineralogical composition, which they subjected to the process of thermal activation and used in concrete mixtures as a substitute for 30% of the cement. The investigated concretes focused on the resistance to sulphate attack, alkali-silica reaction, chloride ingress as well as freezing-thawing resistance and carbonation. They revealed that calcined clays in concrete showed significantly lower pozzolanic activity than in other cement systems, which had a direct impact on the durability of concrete. As far as resistance to chloride ingress is concerned, all clays have passed the test. At the other extreme is freeze-thaw resistance, where only concrete with metakaolin showed satisfactory performance. Carbonation of concrete with calcined clay, due to the reduction of Ca(OH)_2_ consumed in the pozzolanic reaction, progressed faster than in concrete with OPC. The confirmation of pozzolanic activity should not be synonymous with the recognition that the tested clay is suitable for the production of cement or concrete, because depending on its composition and quality its influence on durability parameters may be diametrically different.

Slightly more optimistic conclusions about the durability of concrete with cement blended with calcined clay were formulated by Pierkes et al. [189]. They prepared a series of concretes using cements with 20% (CEM II/A-Q) and 40% (CEM IV/A-Q) of various calcined clays and determined their durability parameters, i.e., resistance to carbonation, chloride migration, freezing-thawing and frost-deicing salts. The first three tests were carried out on non-air-entrained concrete and the fourth on air-entrained concrete. The results indicated that concrete with the addition of calcined clays was able to achieve comparable values of durability parameters as concrete with the addition of other pozzolanic additives, such as fly ash or silica fume. 

Shah et al. [190] prepared a short literature review on the durability of concrete with low clinker content, which is replaced by various mineral additives: fly ash, calcined clay, limestone and slag. This review covered chloride migration/penetration, carbonation, sulphate attack and alkali-silica reaction. The authors concluded that significant chloride resistance can be achieved by using a combination of mineral additives and, after that, the resistance was higher than blended cements. The influence of mineral additions on carbonation was summarised by the statement that above a certain level of exchange rate resistance of cement with mineral additions this resistance started to decrease. Calcined clays were found to cause the faster front of carbonation in the material. ASR (alkali-silica reaction) could be mitigated by SCMs, of which metakaolin is directly named. When it comes to resistance to sulphate attack, the authors are of the opinion that a combination of calcined clay and limestone appears to deteriorate in a sulphate environment.

Castillo Lara et al. [191] have conducted tests of physical-mechanical and durability properties on micro-concrete. From this work only the part concerning the tests of durability, which in this case were water absorption and sorptivity, was discussed. The tests were carried out on a cement system defined as micro-concrete, whose characteristic feature was a maximum aggregate grain size of 5 mm, i.e., more than for mortars and less than for ordinary concrete. Two kinds of calcined clay obtained from raw clay soil were used as a substitute for 30% cement. The results showed a decrease in both water absorption and sorptivity of the micro-concrete tested after using a cement blend with calcined clay compared to the material with OPC only.

The issue of carbonation of binders with metakaolin has been investigated by Bucher et al. [192]. The authors have carried out the research using the cements of type CEM I, CEM II/A-LL and CEM II/A-V as a reference. The depth of carbonation was determined on concrete specimens made with cements without additives and with partial replacement of cement with metakaolin. The results confirmed that the use of calcined clay accelerated the carbonation of concrete except when the component of cement blend was limestone (i.e., in the case of CEM II/A-L cement). Concrete with this blend containing 15% of metakaolin showed higher resistance to carbonation than concrete with Portland cement itself. 

Studies [193,194] were devoted to resistance of cement mortars, containing, e.g., calcined clays, to chloride ingress. The first one discusses the results of tests carried out on mortar specimens prepared using ten different binders. These binders were composed by replacing 35% of the cement with pozzolanic active materials, including calcined clay. The research concerned concretes subjected to an artificial marine environment for 270 days. The use of alternative binders allowed the obtaining of a material with adequate resistance to chloride ingress while reducing CO_2_ emissions by 15%. In the other paper the authors investigated, among others, the resistance to chloride ingress of mortar prepared from cement with 35% content of calcined clay. In one of the mortar series, water was replaced with 0.5 M Na_2_SO_4_ solution, which served as a chemical activator and precursor of ettringite formation. The results of strength and resistance to chloride ingress revealed that the use of such a chemical activator positively affected both parameters.

Calcined clay is a pozzolanic material which makes it possible to reduce the negative effects of alkali-silica reaction, which has been demonstrated in a number of studies [188,195,196]. This is one of the methods of preventing ASR, besides the use of low-reactivity aggregate [197]. Paper [195] presents results of tests of mortars made with high alkali Portland limestone cement, in which 0%–30% of cement binder was replaced with calcined clay. Cement used in the research was characterized by 4.32% Na_2_O_eq_. As the amount of calcined clay in cement increased, the expansion of mortar bars decreased. The lowest expansion value was achieved by mortar with 25% content. The authors attributed this to the formation of more CaSi_2_O_5_ and the associated reduction of sodium silicate, which is the main product of the ASR reaction responsible for the damage caused by it. In [196] the authors presented the results of research on effectiveness of calcined clays in ASR mitigation. Mortars containing highly reactive aggregate and four different types of calcined clays (replacing cement in the amount of 5%, 10%, 15% and 20%) were used in the research. The research showed that the chemical and mineral composition of the clay was crucial for the effectiveness of ASR mitigation. Trümer and Ludwig [188] tested the resistance of concrete with cement mixed with calcined clay to both ASR and sulphate attack. Three calcined clays containing as basic minerals, respectively, kaolinite, illite and montmorillonite after mixing with CEM I cement in a ratio of 30:70 significantly reduced the expansion of mortars and increased their resistance to sulphate attack.

Studies [198,199,200] were devoted to research on the resistance of cement composites to sulphate attack. Aramburo et al. [198] tested cement with a high content of calcined clay to show that it was possible to produce CEM IV/A-SR and CEM IV/B-SR type cements, which complied with the requirements of the current European standard UNE EN 197-1:2011 [201]. The specificity of this study was the very high degree of cement exchange for calcined clay, which was 40%, 50%, 60% and 70%. Apart from calcined clay, two different Portland cements were used in cement mixes, one with high C_3_A content and the other with high C_3_S content. The research showed the required increase in resistance to sulphate cements with calcined clay was accompanied by a decrease in compressive strength. Al-Akhras [199] presented the results of a wide range of research, in which the influence of the exchange of 5%, 10% and 15% of cement to metakaolin on the durability of concrete to sulphate attack was analysed. Apart from various degrees of cement exchange to calcined kaolin, the w/c ratio, initial moist curing period, curing type and air content were also variable parameters. The results allowed to formulate a conclusion that 10% and 15% of the exchange of cement to metakaolin allowed to achieve excellent durability to sulphate attack. Concrete with a lower w/c ratio achieved better durability performances, the time of moist curing proved to be insignificant, autoclaving increased the resistance to moist curing ratio as well as offer a higher air content in concrete. Shi et al. [200] presented the results of a study on the influence of 35% of cement exchange on calcined clay (metakaolin or calcined montmorillonite) or calcined clay and limestone in different proportions on sulphate resistance of mortars. White Portland cement and ordinary Portland cement were used as base cements. The results showed that all mixtures in which the ratio of calcined clay to the sum of calcined clay and limestone was greater than 0.5 showed excellent resistance to sulphate attack.

Quite a large group of papers on durability are focused on cement systems with calcined kaolin. Saillio et al. [202], as well as Yazıcı [203], have analysed various aspects of the durability of concrete and mortars produced with metakaolin addition. Tafraoui et al. [204] gave a brief literature review on the durability of ultra-high performance concrete containing metakaolin. Badogiannis and Tsivilis [205] dedicated their article to the study of the effect of Greek low kaolinite on the durability of concrete, and Shekarchi et al. [206] examined the transport properties (i.e., water penetration, gas permeability, water absorption, electrical resistivity and chloride ingress) of metakaolin-blended cement.

Shi et al. [207] analysed the results of a durability study of Portland cement blends containing, apart from cement, the following additives: pure limestone, pure metakaolin, metakaolin and limestone in 3:1 mass proportion, metakaolin and silica fume, and the three additions simultaneously. The obtained results allowed to state that mortar with pure limestone showed the worst durability parameters in all tests. Mortar with pure cement had the highest resistance to carbonation, but it did not perform well in resistance tests to sulphate attack and chloride ingress, and mortar with pure metakaolinite obtained exactly the opposite resistance parameters.

The issues of concrete durability with calcined clay have also been addressed in two review articles on calcined clays [47] and SCMs in general [58]. 

A review of studies on the durability of concrete with calcined clay makes it clear that the subject is by no means exhausted. There are relatively few studies on frost resistance and resistance to surface scaling. These issues are important for countries where, during the colder seasons, the temperature can repeatedly pass through zero, while relatively fewer articles come from these countries. When studying these durability properties, it also seems appropriate to determine the compatibility of binders containing calcined clay with air-entraining agents. 

Due to the confirmed effectiveness of the addition of calcined clay in limiting the alkali-silica reaction, it is worth considering the simultaneous use of these co-binders with waste glass, either as an aggregate in concrete or as an additional material exhibiting pozzolanic activity. The use of waste glass has so far been limited by concerns about the durability of concrete. Among the studies on the durability aspects of concrete with calcined clays, it is also difficult to find those concerning air permeability. It can be assumed that in this aspect of concrete with the addition of calcined clay will be superior to concrete based on traditional cements, but there is a great deal of room for research between the conjecture and hard evidence.

## 8. Conclusions

This review paper presents the idea of the replacement of Portland cement by addition of calcined clays. Such application of this kind of pozzolanic materials, as clinker/cement replacement or as supplementary cementitious materials, can be an ecological and economically justified way to meet the global need to reduce CO_2_ emissions in concrete technology. Although clay is a material with a very diverse mineralogical composition, its low price, availability and, above all, its distribution in the world make it a valuable supplementary cementitious material in concrete technology. 

Several studies have shown that the thermal treatment is necessary to activate the clay’s minerals or to increase their pozzolanic activity. To determine the conditions of success of the calcination process, the effects of time, temperature and particle size were investigated.

It was shown that mixing various clays in appropriate proportions with cement allowed to obtain a concrete with satisfactory mechanical parameters, much better than the reference one without calcined clay. It was described that the use of calcined clay increased both early age and long-term mechanical properties of cement paste, mortar and concrete. Moreover, it was found that the replacement of cement content by calcined clay influenced the reduction of the bleeding and shrinkage. The simultaneous use of calcined clays as binder components with other types of pozzolan was also shown. 

Research on various aspects of a new binder type—lime calcined clay cement, LC^3^—was thoroughly described. Its composition (Portland clinker, calcined clay, calcium carbonate and gypsum) and as well as fresh mix properties and concrete resistance against aggressive liquid and gaseous media were presented. 

It is confirmed that cement systems containing calcined clay, also with the addition of limestone, showed better durability and increased resistance to most of the aggressive actions to which concrete was exposed. The exception was carbonation, but satisfactory results have also been achieved in this area.

Most of the discussed papers were carried out on mortar or cement paste, and clearly less on concrete. It seems that the application of calcined clay as a supplementary cementitious material in concrete technology requires more research, as not all the findings performed on a smaller scale (i.e., on mortars and cement paste) can be directly transferred to the parameters and performance of concrete.

Future research aimed at improving the long-term durability of cement-based composites containing calcined clays is needed. It is suggested to optimize the composition of calcined clay cements by selecting the appropriate chemical and mineral components of raw materials to obtain the required mechanical parameters. Additionally, more research should be carried out on the practical application in the cement and concrete production of calcined clays of group 2:1. While there is a great deal of basic research on clays and minerals themselves, application research in this field is dominated by clays containing mainly kaolinite.
